# Acute Dietary Zinc Deficiency Impairs Sperm Motility but Not Gonadal mRNA Methylation in Mice

**DOI:** 10.1002/mrd.70144

**Published:** 2026-08-23

**Authors:** Ngozi O. Ibadin, Francisco J. Diaz

**Affiliations:** ^1^ Integrative and Biomedical Physiology Graduate Program, Department of Animal Science, Huck Institute of Life Sciences Pennsylvania State University University Park Pennsylvania USA

**Keywords:** methylation, motility, sperm, zinc

## Abstract

Zinc deficiency is associated with impaired male fertility and with reduced DNA and histone methylation in oocytes, but the temporal relationship between acute dietary zinc deficiency and sperm function is unclear. To fill in these gaps, the effects of acute dietary zinc deficiency on sperm function were tested. Five‐week‐old F1 (DBA × C57BL/6) male and female mice were fed a control diet (29 mg Zn/kg) or zinc‐deficient diet (< 1 mg Zn/kg) for 6 days (*n* = 11/group), a duration selected to model acute zinc depletion based on previous studies demonstrating rapid reproductive defects within a similar timeframe (Tian and Diaz 2012). Sperm number and motility were evaluated by computer‐assisted sperm analysis in all animals (*n* = 11), and testicular histology and DDX4 and YBX2 (MSY2) immunostaining and global m6A levels in testes and liver were measured in a subset of males (*n* = 6) and in ovaries and liver in females (*n* = 6) by an ELISA‐based assay. Zinc deficiency did not alter body weight but significantly reduced total sperm number recovered from the cauda epididymis and impaired total and progressive motility, straight‐line velocity, and hyperactivation. Testes from zinc‐deficient males showed reduced thickness of germinal epithelium and increased basement membrane thickness, disorganized seminiferous tubules, and fewer DDX4‐positive germ cells, while YBX2‐positive cells were largely preserved. Despite these structural and functional defects, zinc deficiency did not significantly change global m6A levels in testes or ovaries, although hepatic m6A methylation was increased in females. These findings demonstrate that a short‐term zinc‐deficient diet rapidly compromises sperm production, motility, and testicular morphology without measurably altering global gonadal mRNA methylation.

## Graphical abstract

Male and female mice were fed a control or zinc‐deficient diet for 6 days. In males, cauda epididymal sperm were analyzed for number, motility, and morphology, and testes were assessed by histology, while m6A mRNA methylation was measured in the testis and liver of males and the ovary and liver of females.

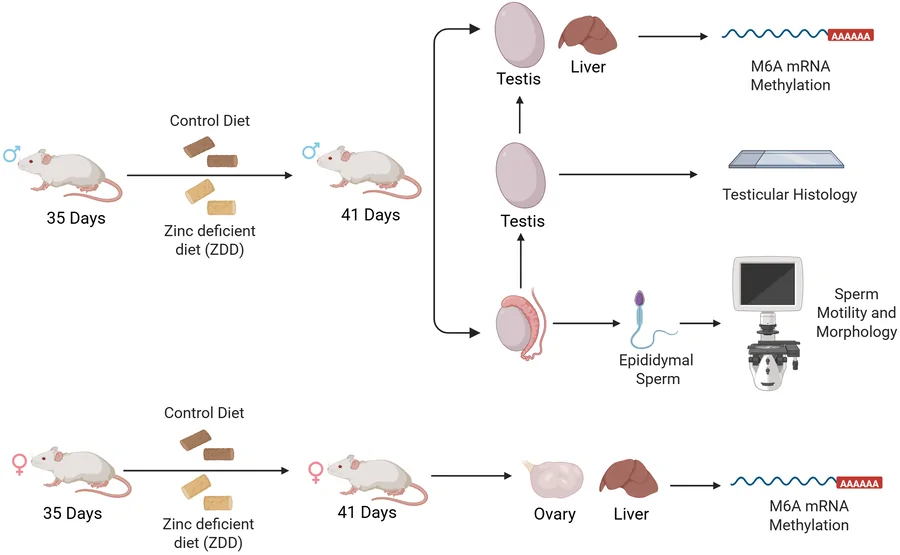

## 1 Introduction

Zinc plays a pivotal role in maintaining male reproductive health, impacting various stages of sperm development and function and ultimately influencing fertility (Fallah et al. 2018; Allouche‐Fitoussi and Breitbart 2020). Zinc is involved in several critical biological processes within the male reproductive system, including sperm formation, maturation, and motility. It modulates androgen synthesis, which is crucial for reproductive processes, emphasizing its importance in male reproductive health (Zhao et al. 2016; Veselinović et al. 2025). Zinc deficiency has been associated with a host of reproductive issues, including reduced testosterone levels (Omu et al. 2015) and increased instances of sperm apoptosis (Beigi Harchegani et al. 2020), but the acute effects of a zinc‐deficient diet (ZDD) on sperm motility and testicular architecture have not been examined in detail. We hypothesize that acute dietary zinc deficiency impairs motility and number in epididymal sperm, which reflects both an acute zinc requirement for sperm motility in the epididymis and progression of germ cells through spermatogenesis in the seminiferous tubule. These effects may be exacerbated by oxidative stress, a key mechanism exacerbated by zinc deficiency, which leads to sperm DNA fragmentation and decreases in sperm membrane integrity, further emphasizing the necessity of zinc for combating oxidative damage and maintaining fertility (Beigi Harchegani et al. 2020).

RNA methylation is a significant epigenetic modification that influences various biological processes without altering the genetic sequence. This modification is crucial in gene expression regulation, genome editing, and cellular differentiation. RNA methylation includes various forms, with N6‐methyladenosine being one of the most abundant and well‐studied (Ruden 2025). RNA N6‐methylation (m6A) plays a significant role in spermatogenesis. It is one of the most prevalent RNA modifications and is crucial throughout the entire process of spermatogenesis, which includes mitosis, meiosis, and spermiogenesis. These modifications impact RNA metabolism by influencing mRNA splicing, translation, and degradation, as well as the biosynthesis and maturity of non‐coding RNAs (Gui and Yuan 2021). Dysregulation of RNA methylation can lead to genomic instability, which is a critical precursor to DNA fragmentation (Wu et al. 2024). Previously, we showed that in newly weaned female mice, acute zinc deficiency lasting 3–5 days significantly reduced female reproductive tract zinc content (31%), compromised ovarian function, and reduced chromatin and DNA methylation of freshly isolated GV oocytes collected immediately after the end of dietary treatments (Tian and Diaz 2012, 2013; Tian et al. 2014, 2017). However, effects on RNA methylation were not evaluated. A secondary aim of this study is to examine the effect of acute zinc deficiency on gonadal RNA methylation. We found that an acute treatment of 6 days on a ZDD impaired sperm motility and reduced sperm number in the epididymis. Consistent with these changes was a reduction in seminiferous tubule density, diameter, and thickness, consistent with impaired spermatogenesis. However, gonadal RNA methylation was not changed but was increased in the female liver. As in females, this work establishes that the testis is susceptible to acute zinc deprivation and requires a near constant supply of zinc to maintain optimal fertility.

## 2 Materials and Methods

### 2.1 Animals

Male and female F1 (DBA × C57BL/6) mice were obtained from the investigators' research colony at the Pennsylvania State University. Mice were housed in the animal facility at Pennsylvania State University under controlled environmental conditions (22°C ± 1°C, 40%–60% humidity) with a 12 h light/12 h dark cycle. Animals were maintained in wire‐bottom polycarbonate cages with ad libitum access to food and water.

Five‐week‐old male mice were randomly assigned to either a control diet or a ZDD and housed individually to prevent coprophagy and cross‐contamination between dietary groups (*n* = 11 mice/group). All animals were euthanized after the dietary treatment period, and tissues were collected immediately for analysis. All procedures were conducted in accordance with the *Guide for the Care and Use of Laboratory Animals* (Institute for Laboratory Animal Research), and all animal use was approved by the Institutional Animal Care and Use Committee (IACUC) at The Pennsylvania State University. The control group was given a control diet (29 mg zinc/kg) based on AIN76 (MP Biomedicals, Solon, Ohio, USA). The ZDD group was given the same diet as the control group, with zinc omitted from the mineral mix (< 1 mg zinc/kg). The ZDD (< 1 mg Zn/kg) and treatment period (6 days) were selected based on established dietary models demonstrated to rapidly reduce systemic and reproductive tissue zinc levels without inducing severe morbidity or weight loss (Tian and Diaz 2012, 2013). This concentration allows induction of acute zinc deficiency within a short timeframe while maintaining animal viability, thereby enabling the assessment of early functional effects on reproductive tissues. All mice were euthanized after 6 days on the diets, and the liver, gonads, and epididymis were collected for subsequent analysis. Epididymal sperm were collected from all animals for testing sperm parameters. In a subset of six animals/group, one testis from each animal and a piece of liver were used for RNA isolation, and the other for histological analyses. Female mice (*n* = 6 group) were treated as described above, and the ovaries and liver were collected for RNA isolation.

### 2.2 Collection of Sperm

Sperm were collected from the cauda epididymis following euthanasia. Each cauda epididymis was placed in 500 all of sperm capacitation media containing 1X Earle's balanced salt solution, sodium pyruvate (0.23 mM), sodium bicarbonate (26.2 mM), 1X essential amino acids, 1X vitamins, EDTA (10 μM), and 3 mg/mL BSA. The cauda epididymis was cut, and sperm were allowed to swim into the capacitation media for 30 min.

### 2.3 Computer‐Assisted Sperm Analysis (CASA)

A CASA (Sperm Class Analyzer [SCA], Microptic S.L., Barcelona, Spain) was employed to evaluate sperm motility and motion kinematics, following previously described methods. In brief, a 10 µL sperm sample was placed on a pre‐warmed counting slide. Using an Olympus BX41 microscope (Olympus Europe GmbH, Hamburg, Germany) with 10× magnification in phase‐contrast mode, images were captured at a frame rate of 60 Hz. A minimum of 10 frames per treatment group, each containing at least 100 sperm, were captured. The SCA system was used to analyze the digitized images. Sperm moving faster than 5 µm/s were classified as motile. The analysis included two sperm quantity measures (concentration and total count) and three gross motility measures: percent motile (MOT), percent progressive motility (PRO), and percent sperm with rapid motility (RAP). MOT encompasses both RAP and PRO. The percentage of rapid sperm is calculated by dividing the number of sperm with time‐average velocity (VAP) ≥ 25 µm/s by the total analyzed sperm count and multiplying by 100. The percentage of progressive sperm is determined by dividing the number of sperm with VAP ≥ 25 µm/s and straightness of trajectory (STR) ≥ 80% by the total analyzed sperm count and multiplying by 100. Seven kinematic measurements were made, as described previously (Palacín et al. 2013). Straight line velocity (VSL) represents the average velocity of a sperm head along the straight line between its initial and final detected positions. Curvilinear velocity (VCL) is the average velocity measured along the actual point‐to‐point path of the cell. VAP measures the sperm head's movement along its spatial average trajectory (a smoothed version of VCL). Linearity (LIN) of the curvilinear trajectory is calculated as VSL/VCL × 100. STR is the LIN of the sperm's average path, calculated as VSL/VAP × 100. The amplitude of lateral head (ALH) displacement represents the maximum lateral displacement of a sperm head from its spatial average trajectory (track width). Sperm hyperactivity was defined based on CASA parameters as sperm exhibiting VCL greater than 271 µm/s, ALH displacement greater than 3.5 µm, and decreased LIN less than 50%, evaluated in at least 200 sperms across a minimum of 10 fields at 60 Hz frame rate.

### 2.4 Sperm Morphology

Sperm morphology was assessed following staining with SpermBlue (Cat # SB‐250‐N, Microptic, Barcelona, Spain), a modified Papanicolaou‐type stain that allows clear visualization of sperm structural features, including the head, midpiece, and tail. Briefly, sperm smears were air‐dried, fixed, and stained according to the manufacturer's protocol, then mounted with a coverslip prior to imaging. Stained sperm cells were imaged under brightfield microscopy at 40× magnification using an Olympus BX41 microscope (Olympus Europe GmbH, Hamburg, Germany), and a minimum of 100 sperm per mouse were evaluated. Images were analyzed using the Morphology module of the SCA software (Microptic, Barcelona, Spain), which automatically detects the sperm head outline via image segmentation and pixel‐based thresholding. For each spermatozoon, the software calculated head area (μm^2^), length, width, perimeter, and derived shape descriptors (ellipticity, elongation, and regularity). Sperm were classified as normal or abnormal using a customized classification scheme configured in the SCA software according to the criteria of Wyrobek and Bruce (Wyrobek and Bruce 1978). Abnormalities were categorized as single or multiple defects according to defect type (e.g., head defects such as amorphous or tapered heads, midpiece defects such as bent or thickened midpieces, and tail defects such as coiled or bent tails), and the proportion of morphologically abnormal sperm was calculated as the percentage of abnormal cells relative to the total number of sperm evaluated per mouse.

### 2.5 Immunohistology

A total of six mice were used for histology and RNA isolation. Mouse testicular tissue from one side of each mouse was placed in cold 4% paraformaldehyde and fixed at 4°C overnight before being transferred to 70% ethanol for preservation; testicular tissue from the other side of each mouse was flash‐frozen for RNA isolation. After gradient dehydration and paraffin embedding, the testicular tissue was sectioned at 5 μm and mounted on slides. Subsequently, the sections were stained with hematoxylin and eosin (H&E) and images taken under brightfield conditions using an Olympus inverted microscope (IX83, Olympus, California, USA) and cellSense imaging software version 4.3. Quantitative morphometric analysis was performed on H&E–stained testicular sections to assess structural changes. Germinal epithelial thickness was measured as the distance between the basement membrane and the luminal surface of the seminiferous epithelium. Seminiferous tubule density was determined as the number of tubules per unit area (mm^2^). Basement membrane thickness was measured at multiple points along the perimeter of individual tubules. For each sample, multiple seminiferous tubules were analyzed, and measurements were averaged to obtain a representative value per animal. All analyses were performed using ImageJ/Fiji software. Spermatogenic index was defined as the proportion of seminiferous tubules containing elongated spermatids, indicating active spermatogenesis. For each sample, seminiferous tubules were examined in H&E‐stained sections, and tubules with visible elongated spermatids were counted as positive. The spermatogenic index was calculated as the percentage of positive tubules relative to the total number of tubules analyzed.

In order to perform germ cell and meiotic cell identification and quantification, testicular sections were labeled with DEAD‐box helicase 4 antibodies (DDX4; Abcam ab13840, Cambridge, UK). To analyze post‐meiotic round spermatids, sections were labeled for YBX2 (NBP2‐19422, Novus Biologicals, Colorado, USA). Briefly, for immunostaining, testicular sections were deparaffinized and rehydrated, followed by antigen retrieval by boiling (15 min) in citrate buffer. After cooling down for 20 min, endogenous peroxidase activity was blocked using 3% hydrogen peroxide for 20 min at RT. Non‐specific binding sites were blocked by incubation with goat serum Blocking Solution for 40 min at RT. Primary antibodies (diluted in blocking buffer) were incubated for 1 h at RT (DDX4 1/200; YBX2 1/100). Afterward, sections were incubated with the secondary antibody Alexa Fluor 488 or Alexa Fluor 594 (Thermo Fisher Scientific, Massachusetts, USA) and mounted with ProLong Gold antifade mounting medium with DAPI (Thermo Fisher Scientific, Massachusetts, USA).

### 2.6 Quantitative Analysis of Post‐Meiotic Germ Cells and Germ Cell Number

Quantitative analysis of seminiferous tubule sections stained for DDX4 or YBX2 was performed using Fiji/ImageJ (version 2.16.0). The fluorescence channel was isolated by splitting multichannel files into individual components. The DDX4 channel was converted to 8‐bit format, and background fluorescence was subtracted to minimize uneven illumination. A consistent global threshold (Moments) was applied to generate a binary mask representing DDX4 or YBX2‐positive cytoplasm. Binary cleaning steps were used to remove noise and ensure continuous cell labeling. For nuclear segmentation, the DAPI channel was subjected to the *Watershed* algorithm to separate touching nuclei. The DDX4 and DAPI binary masks were combined to ensure that only DDX4‐positive regions associated with a nucleus were counted as individual germ cells. Randomly, three seminiferous tubules were manually outlined, and each tubule was saved as a region of interest (ROI) in the ROI Manager. Within each ROI, DDX4 or YBX2‐positive germ cells were then quantified.

### 2.7 RNA Isolation

Testes, liver, and ovarian tissues were collected and processed for total RNA extraction. Tissues were flash‐frozen in liquid nitrogen and then stored at −80°C until processing. To isolate RNA, tissues were thawed and ~10–20 mg of tissue was homogenized in RNA lysis buffer using an electric tissue homogenizer, RNA was isolated from tissue lysate using Quick‐RNA Miniprep cat no# R1055 (Zymo Research, California, USA) according to the manufacturer's instructions, the same RNA isolation protocol was applied to all tissues (testes, liver, and ovaries). Briefly, tissue homogenization was performed using the proprietary RNA lysis buffer provided in the kit containing the detergent N‐Lauroylsarcosine for cell lysis and guanidinium thiocyanate for RNAase inactivation; several steps of centrifugation at 10,000*g* for 30 s in a spin‐away filter were done to remove genomic DNA, flowthroughs were collected in a collection tube and treated with 100% ethanol. DNase treatment was done, followed by several steps of RNA preparation and RNA wash. RNA was collected and stored in 100 μL of DNase/RNase‐free water. RNA concentration and purity were then measured using a NanoDrop device.

### 2.8 m6A RNA Methylation Assay

The amount of m6A methylated RNA was determined using the EpiQuik m6A RNA Methylation Quantification Kit (EpigenTek, Farmingdale, NY, USA). In brief, buffers and solutions, including 1X wash buffer and working solutions of capture (CA) and detection (DA) antibodies, enhancer solution (ES), and positive control (PC) containing 100% m6A oligos, were prepared as proposed by the manufacturer. The extracted RNA was mixed with 80 μL of binding solution, 2 μL of negative control (NC; 100 μg/mL, without m6A RNA), or 2 μL of diluted PC, followed by 50 μL of diluted CA. After a 1‐h incubation at 37°C, the fixed RNA underwent three washes with wash buffer. Next, 50 μL of working DA solution was supplemented per well for a 30‐min incubation at ambient. After washing four times with wash buffer, each well was treated with 50 μL of diluted ES solution at ambient temperature for 30 min. The samples underwent five washes with wash buffer, and the developer solution was added for a 10‐min incubation at ambient temperature, shielded from light. The stop solution (100 μL/well) was added. Absorbance measurement was performed in a 96‐well microplate (Corning, New York, USA, Cat. #3370) at 450 nm using the BioTek Cytation 5 plate reader (Agilent BioTek, California, USA, Cat No BTCYT5FV) within 10 min of adding the stop solution. The m6A methylation rate in each sample was calculated using a standard curve generated from standard solutions provided in the EpiQuik m6A RNA Methylation Quantification Kit (EpigenTek). The relative quantification method was employed to determine the percentages of m6A quantified in different RNA samples relative to the PC RNA.m6A % = [(Sample OD − NC OD) ÷ S]/[(PC OD − NC OD) ÷ P] × 100%, where OD (optical density) represents the absorbance measured at 450 nm, S is the RNA sample input (ng), and PC and NC are the input (ng).

### 2.9 Statistical Analysis

Data was tested for homogeneity of variance. For the data that satisfy the homogeneity of variance, for each sex, one‐way ANOVA was used for comparison between multiple groups. After the ANOVA, the Tukey Honestly Significant Difference post hoc test was used for pairwise comparisons between groups. To examine if ZDD influences bodyweight in male mice or tissue m6A in male and female mice, two‐way ANOVA was used. For sperm quality, Welch's *t*‐test was used to compare the means of the ZDD and control diet groups. The significance level is set at *α* = 0.05. All statistical analyses were performed using the GraphPad Prism 10.60 statistical software (GraphPad Software, San Diego, California, USA).

## 3 Results

### 3.1 Effects of Acute Dietary Zinc Deficiency on Epididymal Sperm Number

**Figure 1:**
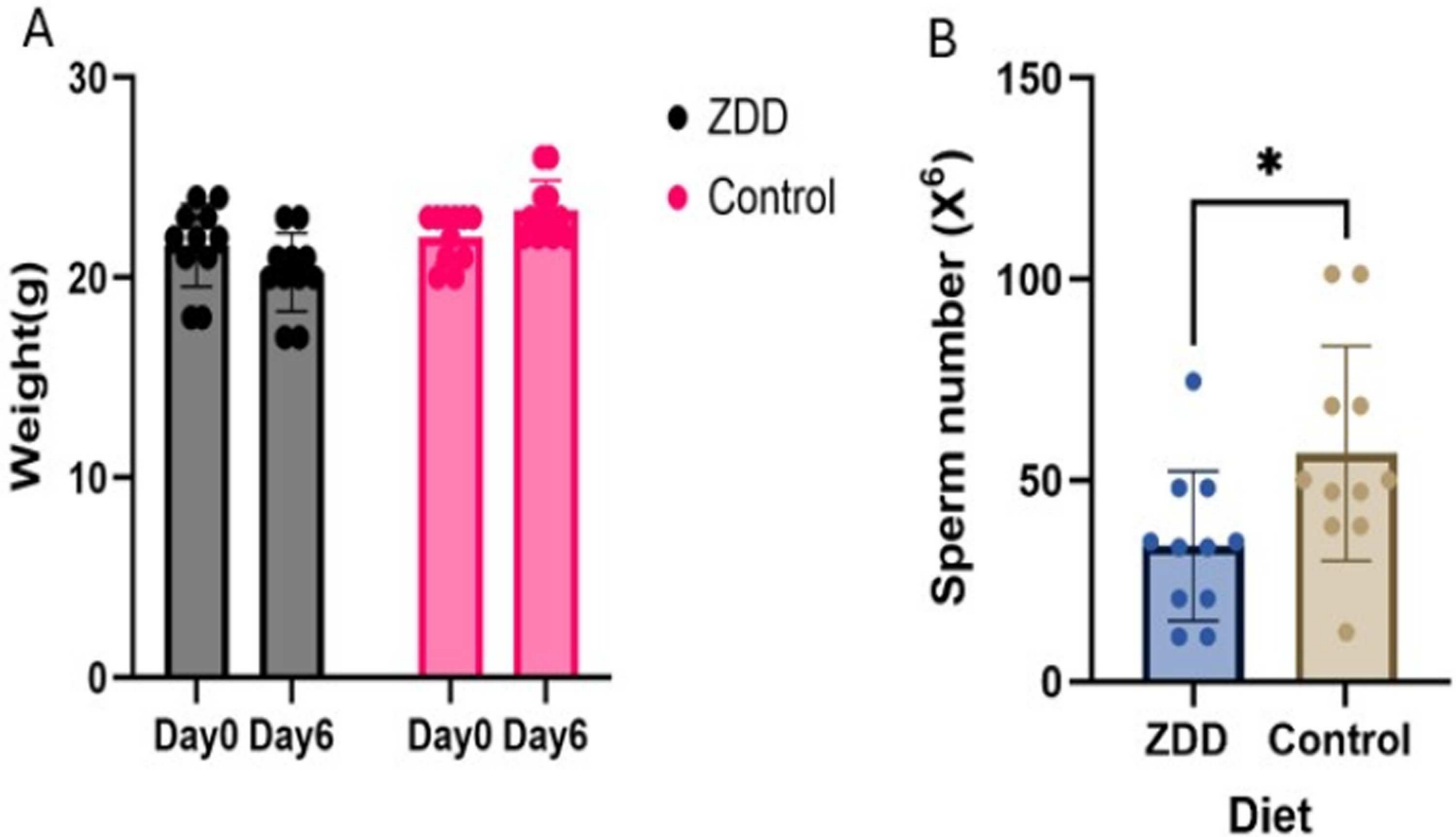
Effects of acute dietary zinc deficiency on male body weight and epididymal sperm number. (A) Body weights on Day 0 and Day 6 in the control and ZDD groups. No difference by two‐way ANOVA (treatment effect *p* = 0.1013, time effect *p* = 0.9770, and treatment × time interaction *p* = 0.1663). (B) Sperm number using Welch's *t*‐test (*p* = 0.0367). Statistical significance set at *p < 0.05. Results were obtained from 11 mice in each diet group.

The effects of acute dietary zinc deficiency on epididymal sperm number and body weight were examined. Figure 1A illustrates the change in body weight from Day 0 to Day 6 for both the ZDD group and the control group. There were no statistical differences between the ZDD and control mice (*p* = 0.9770). Sperm number was assessed at a 1:10 dilution for both groups (Figure 1B). Total sperm recovered from the cauda epididymis was significantly lower in ZDD mice compared to controls (*p* = 0.0367). The mean difference in sperm number between the two groups was 23.96 ± 10.47 × 10^6^ sperm cells. This finding suggests that acute dietary zinc deficiency has a rapid impact on sperm production and may affect sperm quality.

### 3.2 Dietary Zinc Deficiency Altered Sperm Motility and Speed

**Figure 2:**
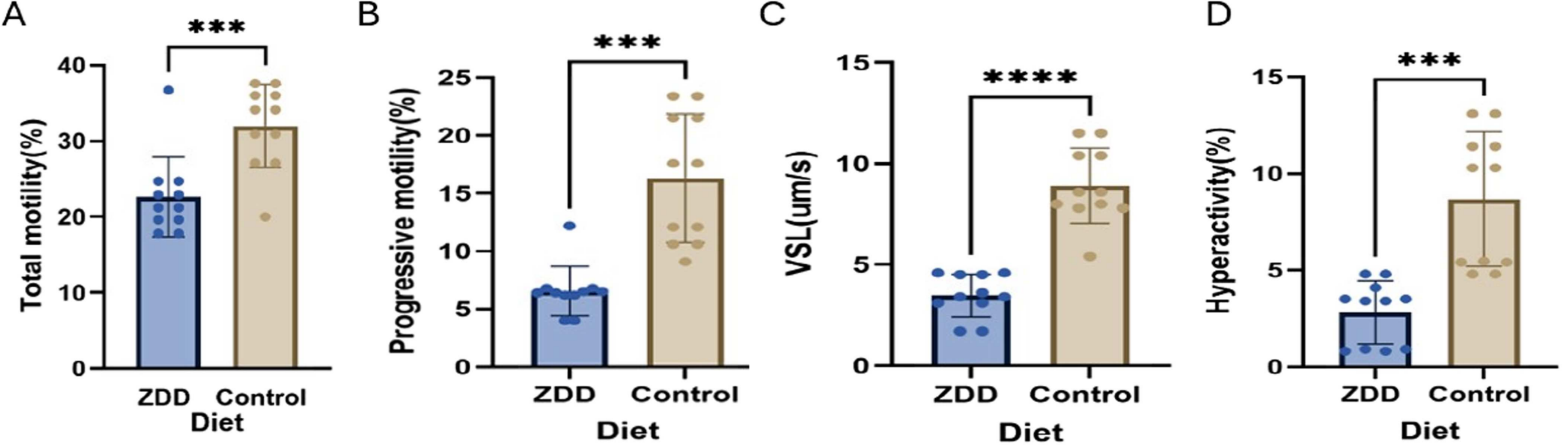
Zinc‐deficient diet (ZDD) impairs sperm motility. Computer‐assisted sperm analysis of sperm motility and head morphology in mice exposed to a ZDD and a control diet. (A) Total motility (%), (B) progressive motility, (C) sperm straight line velocity (μm/s), and (D) sperm hyperactivity (%). Statistical significance set at ***p < 0.05; ****p < 0.0001. Results were obtained from 11 mice in each diet group.

Acute dietary zinc deficiency significantly altered sperm quality parameters in mice. Sperm motility and morphology were assessed using CASA and analyzed using the SCA software (Microptic, Barcelona, Spain) following exposure to ZDD or a control diet. Total sperm motility was significantly reduced in the ZDD group (22.65 ± 2.284) compared to the control group (31.95 ± 2.284; *p* = 0.0006, Figure 2A). The progressive motility of sperm, a measure of forward movement, decreased by 59.2% in the ZDD group (*p* < 0.0001, Figure 2B). Sperm velocity was also affected by zinc deficiency. The straight‐line velocity, which quantifies sperm speed, was reduced in the ZDD group (3.473 ± 0.6448) compared to the control group (8.909 ± 0.6448; *p* < 0.0001, Figure 2C). To evaluate the impact of acute dietary zinc deficiency on sperm capacitation, we measured sperm hyperactivity. The ZDD group exhibited a significant reduction (67.6%) in sperm hyperactivity compared to the control group (*p* < 0.0001, Figure 2D).

**Figure 3:**
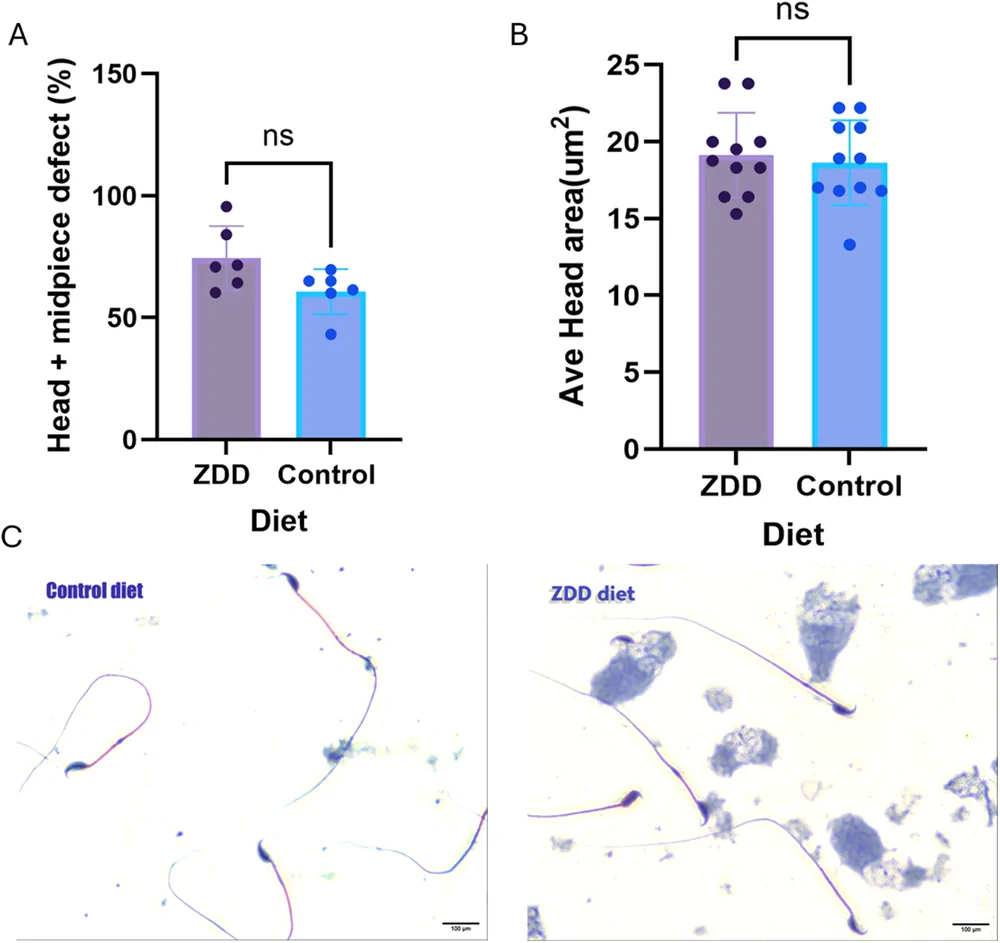
Zinc‐deficient diet (ZDD) does not alter sperm morphology. (A) sperm head and midpiece defects, (B) sperm head area, and (C) representative images of sperm cells in the control group and ZDD group. Results were obtained from 11 mice in each diet group.

The assessment of sperm quality extended beyond motility parameters to include an evaluation of sperm morphological abnormalities, specifically focusing on the sperm head and midpiece defects following staining with SpermBlue (Microptic, Barcelona, Spain). Sperm morphology analysis did not reveal any statistical difference between the ZDD group and control diet group for head/midpiece defects and sperm head area (Figure 3).

### 3.3 Testicular Tissue of Zinc Deficient Mice Showed Morphological Alterations

**Figure 4:**
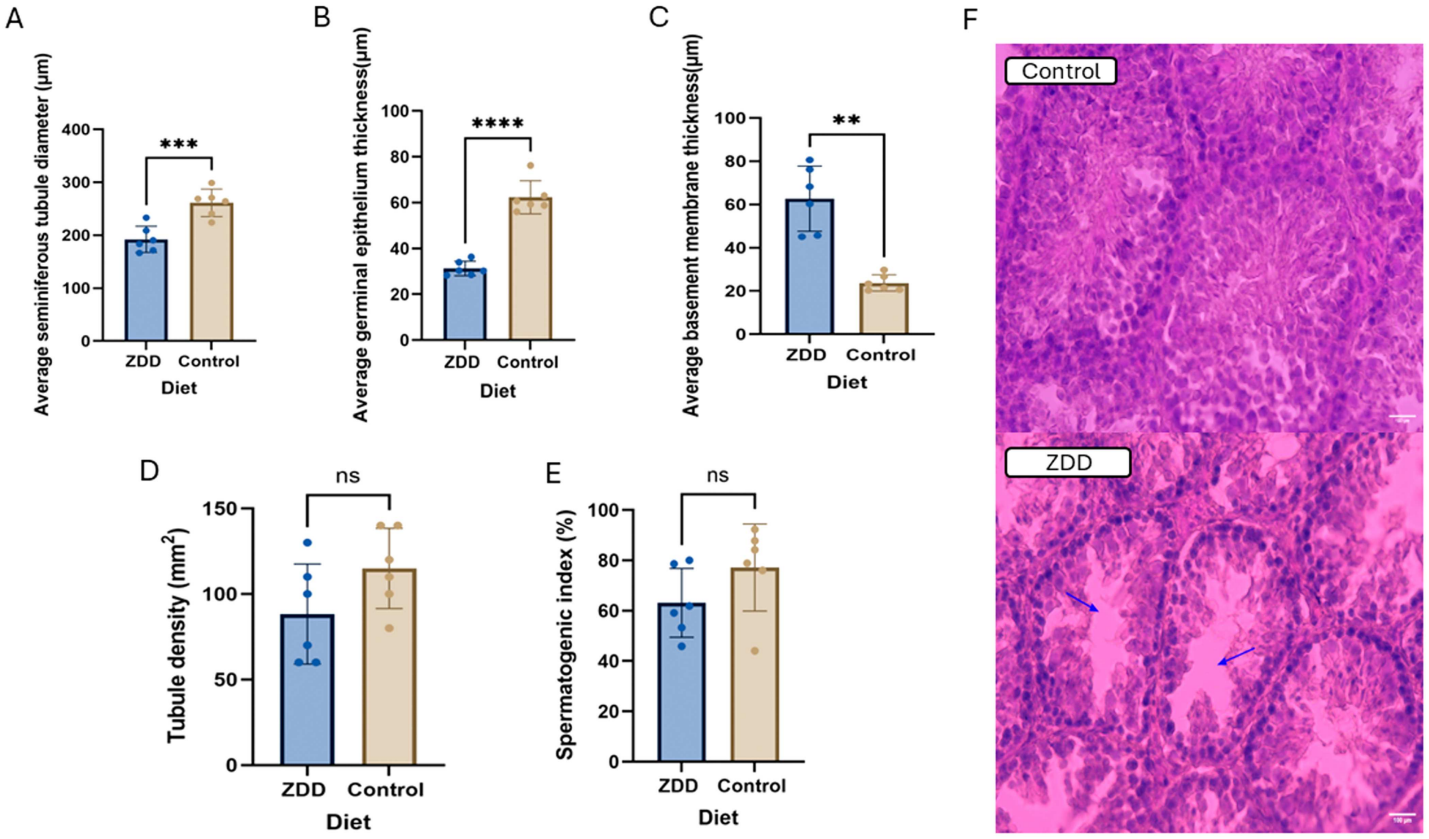
Effects of dietary zinc deficiency on testicular morphology. (A) Average seminiferous tubule diameter of spherical tubules, (B) average germinal epithelial thickness, (C) average basement membrane thickness, (D) seminiferous tubule density (number of tubules per mm^2^), and (E) spermatogenic index (proportion of seminiferous tubules with elongated spermatids) in testes from control or ZDD. (F) Representative images of H&E‐stained testicular tissue (blue arrow: wide tubular spaces with thin germinal epithelium). Statistical significance set at ****p < 0.0001, ***p < 0.009, **p < 0.0011.

Histological examination of testicular tissue from ZDD mice revealed pronounced morphological alterations of the seminiferous tubules. H&E staining demonstrated a significant reduction in the diameter of seminiferous tubules in the ZDD group (192.3 ± 14.73) compared to the control group (261.2 ± 14.73), indicating compromised tubular structure (Figure 4A). Quantitative analysis further showed a marked decrease of 49.8% in the thickness of the germinal epithelium in zinc‐deficient mice relative to controls (Figure 4B, *p* < 0.0001). Additionally, the basement membrane of the seminiferous tubules was significantly thicker in the ZDD group (62.74 ± 7.158) compared to the control group (24.15 ± 7.158; *p* < 0.0011, Figure 4C). However, tubule density per square millimeter did not differ significantly between groups (*p* = 0.1136, Figure 4D).

Functional assessment of spermatogenesis through the spermatogenic index, which measures the proportion of tubules containing elongated spermatids, revealed no significant difference between ZDD and control mice (*p* = 0.1508, Figure 4E). Qualitative evaluation of testicular sections from zinc‐deficient mice highlighted distorted and less compact seminiferous tubule architecture, characterized by expanded luminal spaces within the tubules (Figure 4F).

### 3.4 Acute Dietary Zinc Deficiency Decreased Germ Cell Number in Seminiferous Tubules

**Figure 5:**
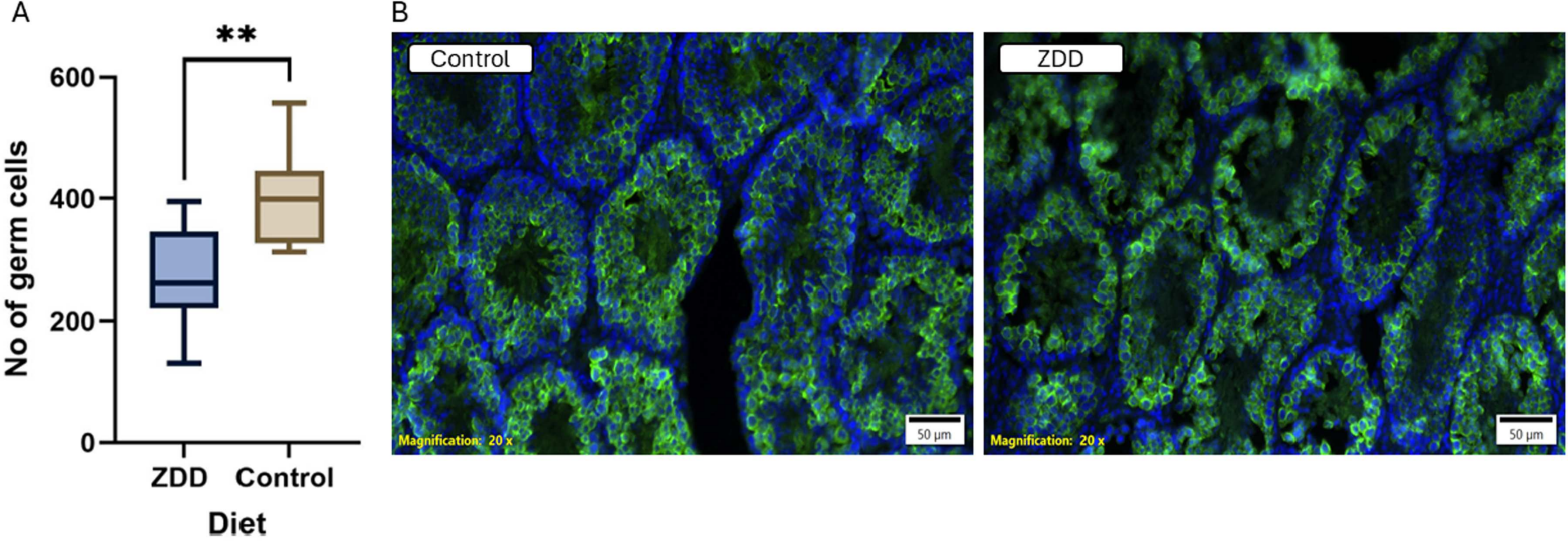
Effect of acute dietary zinc deficiency on germ cell number. (A) Number of germ cells in control and ZDD testes stained with DAPI (DNA, blue) and DDX4 (green) to mark the germ cells (*p* = 0.0026). (B) Representative images of control and ZDD testes. Scale bar = 50 μm. Statistical significance set at **p < 0.05.

**Figure 6:**
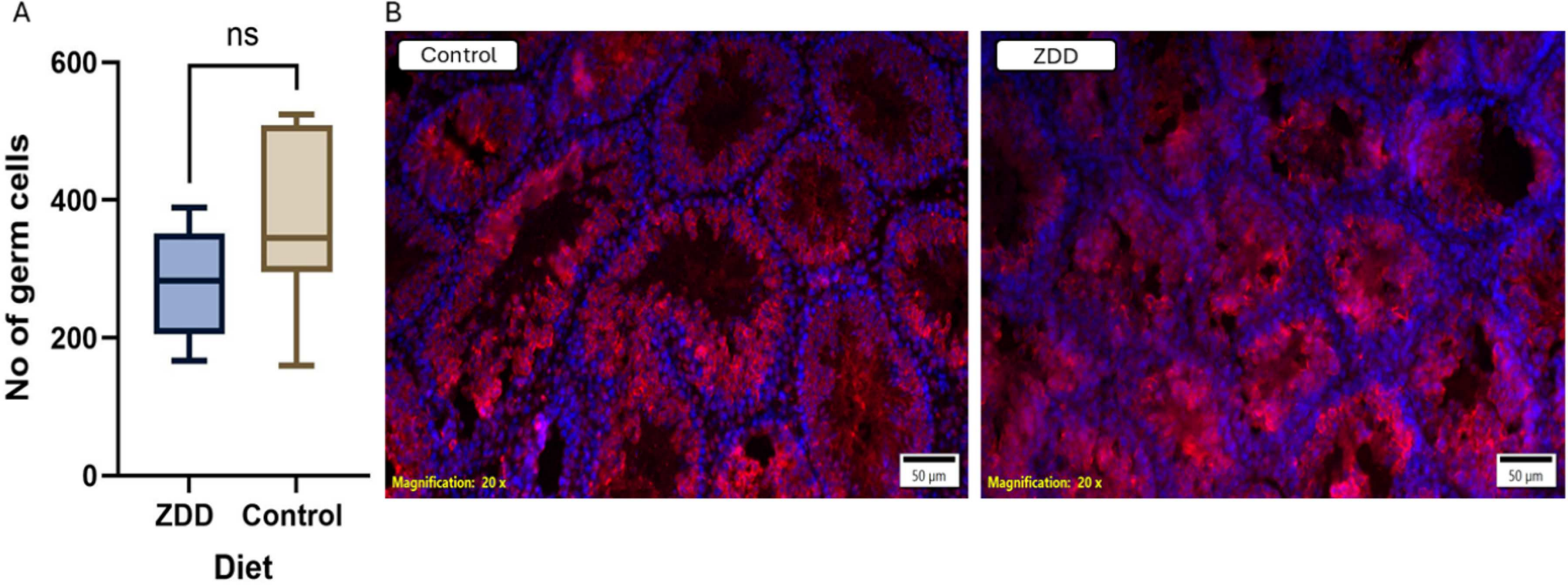
Effect of acute dietary zinc deficiency on post‐meiotic germ cell number. (A) Number of post‐meiotic germ cells in control and ZDD testes stained with DAPI (DNA, blue) and YBX2 (red) to mark diplotene germ cells. (B) Representative images of control and ZDD testes. Scale bar = 50 μm.

Consistent with a reduction in epididymal sperm, acute dietary zinc deficiency significantly decreased germ cell number in mouse testes by 31.4% (Figure 5, *p* = 0.0026), as demonstrated by immunohistochemical analysis using a total germ cell‐specific marker, DDX4. Further assessment of post‐meiotic early spermatids using the marker YBX2 showed a decrease (*p* = 0.0588) in cell number in the zinc‐deficient group (282.2 ± 44.13) compared to the control group (372.3 ± 44.13, Figure 6). There was a 24.2% decrease in the number of YBX2‐positive cells in ZDD males compared to control; however, this did not reach statistical significance.

### 3.5 Impact of Zinc Deficiency on N6‐Methyladenosine RNA Methylation in Liver and Gonadal Tissues

**Figure 7:**
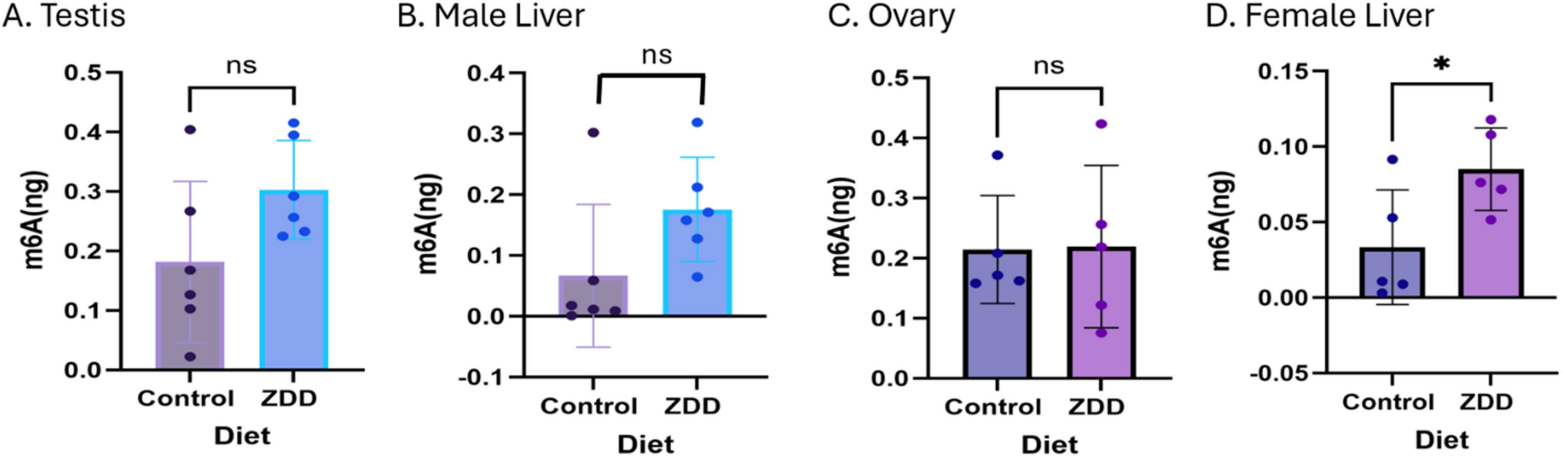
N6‐methyladenosine RNA methylation levels in (A) testes, (B) male liver, (C) ovary, and (D) female liver. Bars represent mean m6A levels, with error bars indicating standard deviation. Asterisk (*) denotes statistically significant difference (*p* < 0.05).

To examine the impact of dietary zinc deficiency on RNA methylation, we extracted RNA from male and female liver and gonads and analyzed for N6 methyladenosine RNA methylation levels. m6A RNA methylation in liver and gonadal tissues revealed distinct patterns between zinc‐deficient and control diet groups across sexes (Figure 7). Testicular m6A levels were higher in the zinc‐deficient group compared to controls; however, this was not statistically significant (Figure 7). Ovarian m6A levels were nearly identical in control and ZDD groups (Figure 7). We also compared m6A levels in liver tissue for both male and female mice. Liver m6A levels were increased in female mice by 57% (*p* = 0.0419), but not in male mice in the ZDD group (Figure 7).

## 4 Discussion

Zinc plays a crucial role in both male and female reproductive systems in various species from invertebrates to humans (Fallah et al. 2018; Allouche‐Fitoussi and Breitbart 2020; Garner et al. 2021). In large animal species, zinc is essential for multiple aspects of male reproductive health, including sperm maturation and maintaining sperm DNA integrity, and contributes to the structure and function of sperm proteins that regulate various stages from spermatogenesis and fertilization (Kerns et al. 2018; Zigo et al. 2022). In humans, chronic zinc deficiency can lead to various reproductive issues, such as increased oxidative stress and apoptosis, contributing to male infertility (Beigi Harchegani et al. 2020). Moreover, seminal plasma zinc levels are often lower in infertile men as compared to fertile counterparts, and zinc supplementation has been shown to improve semen volume and morphology (Zhao et al. 2016). In female mice, acute zinc deficiency for 3–5 days impairs oocyte quality, including reduced maturation and fertilization, and reduced developmental potential of subsequent embryos, likely due to alteration of oocyte chromatin methylation (Tian and Diaz 2012, 2013; Tian et al. 2014) and leads to a 31% reduction in tissue zinc concentrations in the reproductive tract (Tian et al. 2017). In the present study, we show that male fertility is also sensitive to acute dietary zinc deficiency in mice.

The main findings from this study were that after only 6 days of ZDD, total sperm number recovered from the cauda epididymis was significantly reduced in ZDD males (*p* = 0.0367). This demonstrates that sperm production is rapidly compromised by acute zinc deprivation. Together with previous human data linking lower seminal zinc to reduced semen volume, motility, and normal morphology (Zhao et al. 2016), these findings support the idea that a continuous dietary supply of zinc is required to sustain optimal sperm production. However, many previous studies in mice have used longer treatments of 2 weeks or more, which would lead to chronic effects including weight loss, increased inflammatory response, and decreased testosterone concentrations (Croxford et al. 2011; Sun et al. 2023; Hou et al. 2025). In our study, animals were only exposed to a brief 6‐day treatment with a ZDD and yet had a pronounced effect on sperm number recovered from the epididymis. The cause of this decrease may involve impaired spermiogenesis or the release of mature sperm from the seminiferous tubules (spermiogenesis) or cell death of sperm cells already in the epididymis. Since the dietary treatment was brief, an effect on earlier stages of spermatogenesis does not explain the reduction in cauda epididymis concentrations. More work is needed to pinpoint the exact cause, but this represents a new finding on the acute role of zinc in maintaining sperm concentrations in the epididymis.

Zinc deficiency also had pronounced effects on sperm quality, particularly motility‐related parameters. Compared to the control group, ZDD sperm had 43.5% reduction in total motility, 59.2% decrease in progressive motility, 60.7% decrease in straight‐line velocity, and 67.8% decrease in sperm hyperactivity, indicating that zinc is essential not only for sperm production but also for the acquisition or maintenance of normal motility and capacitation. Morphology of the sperm head did not appear to be affected by treatment since sperm head area did not differ between groups (Figure 3). However, motility defects emerge rapidly, consistent with the notion that zinc‐dependent enzymes, ion channels, and signaling pathways that support energy metabolism, flagellar activity, and membrane remodeling are particularly vulnerable to acute zinc deprivation (Yamaguchi et al. 2009; Omu et al. 2015).

Although sperm morphology did not differ significantly, the morphology of the seminiferous tubules was altered. Zinc deficiency resulted in significant structural alterations within the seminiferous tubules, including a marked reduction in germinal epithelial thickness, suggesting loss of germ cells and impaired spermatogenic activity. A concurrent decrease in basement membrane thickness further indicates compromised structural support of the seminiferous tubules under zinc‐deficient conditions. In contrast, seminiferous tubule density remained unchanged, indicating that the overall number of tubules was preserved despite pronounced cellular and structural disruption. These changes are compatible with early germ cell loss and architectural weakening of the seminiferous tubules. The spermatogenic index, defined as the proportion of tubules containing elongated spermatids, did not differ significantly between groups, indicating that mature spermatids were still present at the time of collection despite possible changes at earlier stages of spermatogenesis. Qualitative observations of less compact tubules with widened luminal spaces further support compromised seminiferous structure. These findings are consistent with previous work in other species showing that more prolonged or severe zinc deficiency leads to reductions in seminiferous tubule diameter and disruption of testis architecture tubules (Martin et al. 1994; Hamdi et al. 1997; Ge et al. 2008), and indicate that such changes can begin to manifest within a few days of zinc depletion.

We also observed acute effects of zinc deficiency on germ cells in the seminiferous tubules. We quantified total germ cells by DDX4 (VASA) immunohistochemistry. DDX4 is an RNA helicase localized in the cytoplasm of germ cells, which plays a crucial role in maintaining germ cell meiosis, facilitating the transition from undifferentiated to differentiated germline states (Kuramochi‐Miyagawa et al. 2010; Hickford et al. 2011). Also, we quantified early spermatid cells by YBX2 staining. The significant reduction in DDX4‐positive cells in ZDD testes (*p* = 0.0026) indicates an overall loss of total germ cells and impaired spermatogenic activity under zinc deprivation. In contrast, YBX2‐positive cells, representing late spermatocytes and round spermatids, were not significantly different. These observations suggest that earlier germ cell stages (before pachytene) may be more sensitive to acute zinc depletion than post‐meiotic stages. It's likely that there was not a decline in post‐meiotic cells because the treatment was too short for a reduction in early germ cells to be reflected in the meiotic and spermatid population. Early germ cells may be more vulnerable to oxidative stress and impaired proliferation associated with zinc loss (Omu et al. 2015), whereas post‐meiotic cells may display greater short‐term resilience (Yamaguchi et al. 2009; Beigi Harchegani et al. 2020). The net effect was a 31.4% reduction in total germ cell number, even though some later‐stage spermatids remain detectable. However, this does not explain the reduction in spermatozoa in the cauda epididymis since there would not be enough time for reductions in pre‐meiotic germ cells to cause reduced spermatozoa formation. This suggests that a process intrinsic to the cauda epididymis is altered by the ZDD, but this will require further work to fully understand.

A second goal of this study was to determine whether acute zinc deficiency reduces global RNA m6A methylation in gonads and liver. This was prompted by our previous study showing reductions in oocyte DNA and chromatin methylation after feeding an acute ZDD for 5 days (Tian and Diaz 2013). Moreover, zinc is a cofactor for numerous enzymes, including some that influence RNA metabolism and epigenetic regulation (Sun et al. 2023). We observed tissue and sex specific effects. There was a surprising 57.6% increase in liver m6A levels in females (*p* = 0.0419), but not in males, on the ZDD compared with controls. This suggests that hepatic RNA methylation is responsive to zinc status in a sex‐dependent manner. The liver is central to nutrient sensing and metabolic homeostasis, and altered m6A patterns may represent an adaptive response to nutritional stress, modulating mRNA stability and translation of genes involved in zinc transport, oxidative defense, and energy metabolism (Ma and Wu 2022; Ming et al. 2024). The absence of a statistically significant effect in the male liver may reflect sex‐specific regulatory mechanisms or differing baseline zinc reserves and hormonal environments between males and females. Although not significant, there were numerical increases in m6A methylation in testes and liver in males. These results could indicate a regulatory role of m6A methylation in RNA metabolism and gene expression, particularly under conditions of stress or nutritional deficiency. In the testes, m6A methylation regulates spermatogenesis and is involved in male fertility. Dysregulation of m6A could lead to altered gene expression and contribute to issues such as infertility (Cai et al. 2021; Ma and Wu 2022; Ming et al. 2024).

In summary, this study demonstrates that even a short‐term reduction in dietary zinc leads to a rapid decline in sperm concentration, impaired motility and hyperactivation, and detectable disruption of testicular architecture and germ cell populations, without measurable changes in global m6A methylation in gonadal tissues. Together with previous work in females, these data support the conclusion that both the ovary and the testis are highly susceptible to acute zinc deprivation and require a near‐constant supply of dietary zinc to maintain optimal fertility.

## Author Contributions

**Ngozi O. Ibadin:** conceptualization, writing – original draft, writing – review and editing, investigation. **Francisco J. Diaz:** conceptualization, funding acquisition, writing – original draft, writing – review and editing.

## Ethics Statement

No human subjects were involved in this study.

## Conflicts of Interest

The authors declare no conflicts of interest.

## Data Availability

All data for this article is available upon reasonable request.
